# Supplementary figures and images for: Mouse B2 SINE elements function as IFN-inducible enhancers
Source: eLife. 2023 May 9;12:e82617. doi: 10.7554/eLife.82617 (PMC10229128; doi:10.7554/eLife.82617)

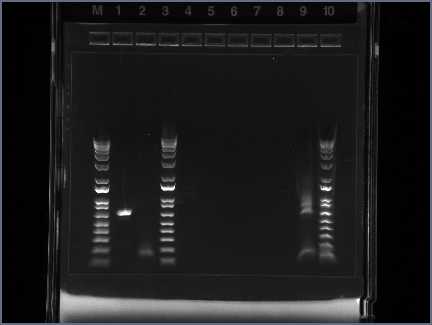

Supplement: Figure 3—figure supplement 4—source data 1. [file elife-82617-fig3-figsupp4-data1.zip › Figure 3-figure supplement 2-source data 1/F3_FS2_B_raw.jpg]

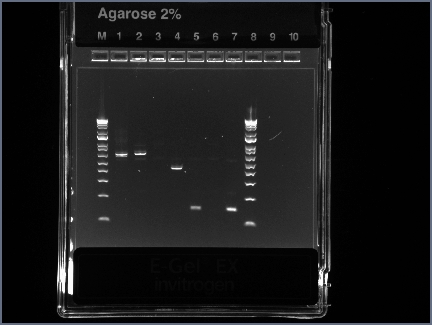

Supplement: Figure 3—figure supplement 4—source data 2. [file elife-82617-fig3-figsupp4-data2.zip › Figure 3-figure supplement 2-source data 2/F3_FS2_D_raw.jpg]

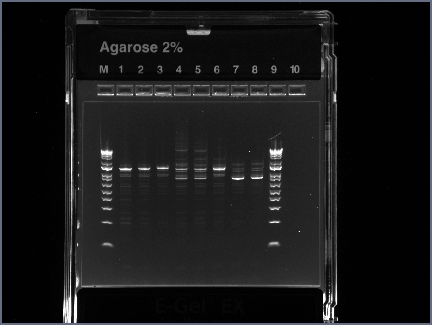

Supplement: Figure 3—figure supplement 4—source data 3. [file elife-82617-fig3-figsupp4-data3.zip › Figure 3-figure supplement 2-source data 3/F3_FS2_E_raw.jpg]

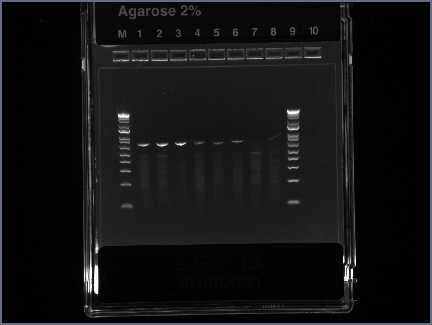

Supplement: Figure 3—figure supplement 4—source data 4. [file elife-82617-fig3-figsupp4-data4.zip › Figure 3-figure supplement 2-source data 4/F3_FS2_F_raw.jpg]

**A**

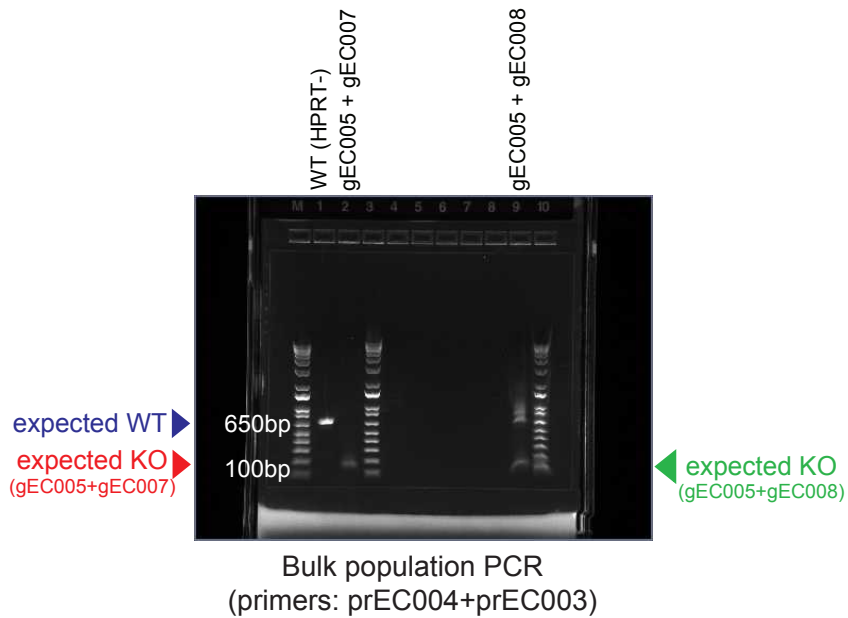

**B**

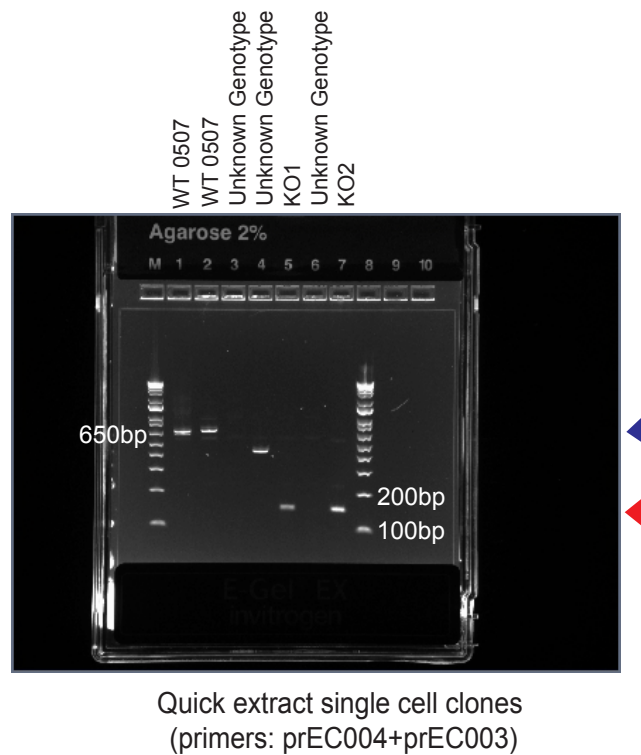

**C**

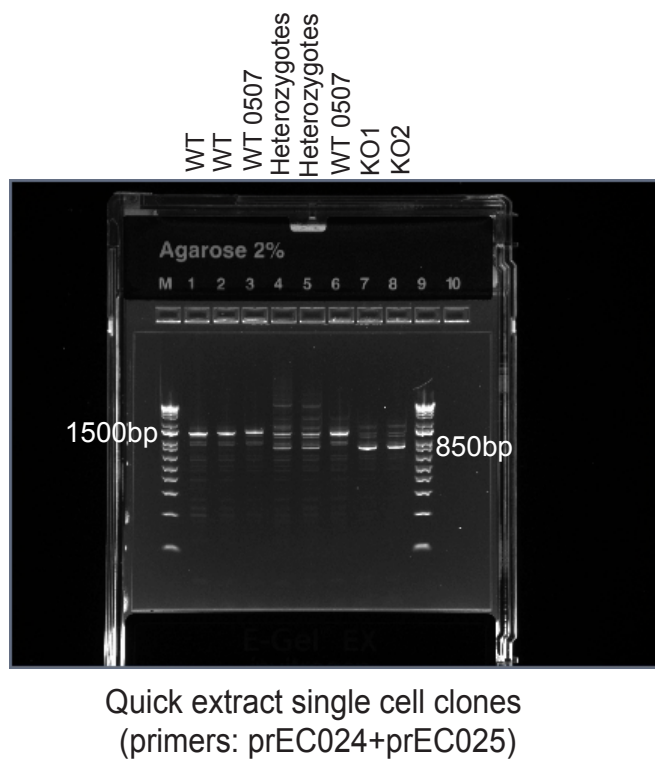

**D**

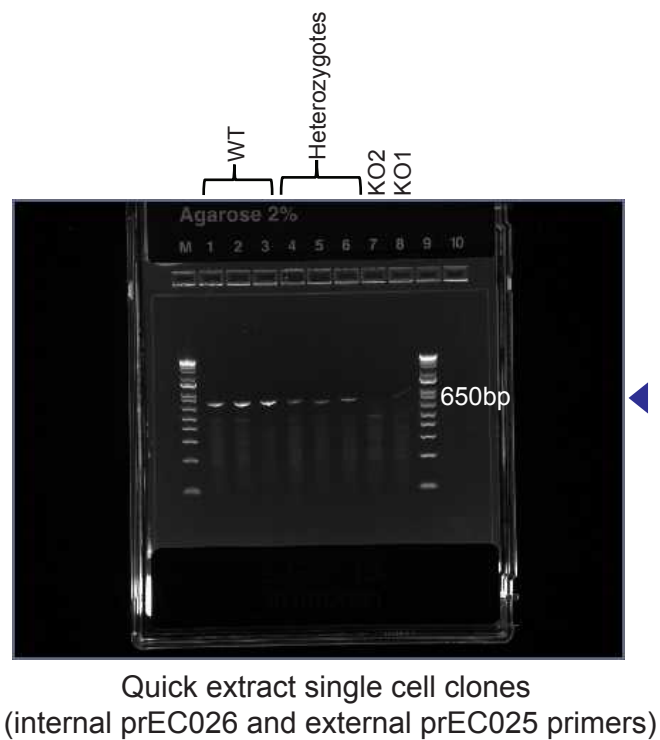

Supplement: Figure 3—figure supplement 4—source data 5. — (A) Uncropped, labeled gel from Figure 3—figure supplement 4B. (B) Uncropped, labeled gel from Figure 3—figure supplement 4D. Unknown genotype indicates the band length did not correspond with either WT, heterozygote, or KO genotype or product from a single cell colony was loaded but no band appeared. (C) Uncropped, labeled gel from Figure 3—figure supplement 4E. (D) Uncropped, labeled gel from Figure 3—figure supplement 4F. [file elife-82617-fig3-figsupp4-data5.zip › Figure 3-figure supplement 2-source data 5/Response_Figure_6.pdf]
